# HISTORICAL CHANGES IN COLLEGE EDUCATION AND EMPLOYMENT: GENDER EFFECTS ON LATE-LIFE COGNITIVE STATUS

**DOI:** 10.1093/geroni/igad104.3390

**Published:** 2023-12-21

**Authors:** Marina Larkina, Jacqui Smith

**Affiliations:** University of Michigan, Ann Arbor, Michigan, United States; University of Michigan, Ann Arbor, Michigan, United States

## Abstract

Early-life education and gender are well-known predictors of cognitive status in old age but multiple mechanisms underlie these associations. Recent US research on late-life cognition and dementia prevalence has highlighted the roles of age-cohort and racial/ethnic differences K-12 education. Less attention has been given to historical increases since the 1970s in the completion of college degrees by women and their subsequent employment histories. We examined new data for two cohorts (N = 9926; 50% born before 1948 vs. Baby Boomers, born 1948-1959) in the Health and Retirement Study (HRS) about the quantity (years, degree types) and content (field of study) of their college degrees. College information and employment histories were collected in a Life History Mail Survey (2015-2017). Cognitive status based on the Langa-Weir algorithm was defined as normal, cognitive impairment not dementia or dementia (CIND/D). Compared to men, women were less likely to have post-high school education (p < .002) or to have worked after their education. Cognitive status was predicted by degree type and work history. Gender by degree and gender by work interactions (for Baby Boomers only) revealed that having a BA/BS degree provided more protection against cognitive impairment for women than for men with the same degree, and women who worked had lower odds ratio of having CIND/D (0.36 [CI 0.17-0.77], p < .01). College majors were gendered and significantly accounted for variance in late-life cognitive status among the college educated. These findings provide new insight into the gendered life histories related to late-life cognition.

